# Cyclic GMP/cGMP-dependent protein kinase type I signaling and cysteine-rich LIM-only protein 4 in AngII/AT_1_R-induced heart hypertrophy and fibrosis

**DOI:** 10.1186/2050-6511-16-S1-A13

**Published:** 2015-09-02

**Authors:** Robert Lukowski, Julia Straubinger, Peter Ruth

**Affiliations:** 1 Department of Pharmacology, Toxicology and Clinical Pharmacy, Institute of Pharmacy, University of Tuebingen, Tuebingen, Germany

## Background

Cardiac hypertrophy is an adaptive response of the heart to many cardio-vascular disorders including hypertension, infarction and defects of the valves. Elevated levels of cardiac cyclic guanosine-3',5'-monophosphate (cGMP) activate cGMP-dependent protein kinase I (cGKI), which reportedly exhibited either anti-fibrotic and anti-hypertrophic effects or did not change the cardiac remodeling response. Based on these conflicting results, we and others suggested that the ability of natriuretic peptides (NP) to oppose detrimental changes via cGMP/cGKI might be strongly influenced by the stress stimuli that actually trigger the harmful events i.e. Angiotensin II (Ang II) modulating multiple reno-vascular and cardiac functions via G q-coupled Ang II type 1 receptors (AT1Rs). It is the overall aim of this study to dissect the molecular details underlying the cross-talk between the NP/cGMP/cGKI pathway and AngII/AT1R signaling in the heart.

## Methods

We investigated the role of cGKI and its substrate cysteine-rich LIM-only protein 4 (CRP4) in neuro-hormonal induced heart hypertrophy upon Angiotensin II (Ang II) infusions (2 mg/kg/d) and in transgenic mice overexpressing the human AT1R specifically in cardiomyocytes ( MHC-AT1Rtg/+). Physiological growth adaption of the heart muscle was studied by healthy exercise training using a duration-controlled swimming protocol in CRP4 wild type (CRP4-WT) and CRP4 knockout (CRP4-KO) animals. The extent of the cardiac growth response was defined by referring changes in heart-weight to body-weight (HW/BW) or tibia length (HW/TL), Sirius Red staining as a quantitative measure of fibrosis and by echocardiographic parameters in CRP4-KO and double-mutant MHC-AT1Rtg/+ x CRP4-KO mice in comparison to age- and littermate CRP4-WT and MHC-AT1Rtg/+ x CRP4-WT mice, respectively. Hypertrophic and fibrotic marker genes, putative effects of Ang II and AT1R overexpression on components of the NP/cGMP/cGKI pathway and the levels of other members of the CRP protein family i.e. CRP1 and the muscle LIM protein CRP3/MLP were analyzed in total mRNA and protein preparations isolated from healthy and hypertrophic ventricles. These experiments were corroborated by investigating the myocardial dynamics of the CRP4 interactome ±Ang II.

## Results

HW/BW and HW/TL ratios between CRP4-KO and CRP4-WT mice did not differ at baseline or upon healthy exercise; however, in response to the Ang II infusions cardiomyocyte size, normalized heart ratios and total cardiac mass as well as the amount of interstitial fibrosis were elevated in CRP4-negative hearts, whereas anti-fibrotic factors such as BNP were lower in the absence of CRP4. Evidence for a protein complex containing cGKI and CRP4 in lysates obtained from hypertrophic CRP4-WT hearts provides a link between cGMP/cGKI and CRP4 in a setting of amplified Ang II signaling. Our ongoing analyses of the MHC-AT1Rtg/+ mouse model, which develops a progressive cardiac remodeling phenotype in the absence of hypertension, identified an increase in basal cGMP levels and high abundance of cardiac cGKI protein confirming activation of the cGMP/cGKI pathway under these conditions. Finally, amplified AT1R signaling in the CM caused a gradual decline in multiple parameters of cardiac function (percent fractional shortening (%FS), ejection fraction (EF) etc.), which were all significantly more distorted in double-mutant MHC-AT1Rtg/+ x CRP4-KOs than in MHC-AT1Rtg/+ x CRP4-WT mice.

## Conclusion

Increased susceptibility of CRP4-deficient hearts to both chronic Ang II exposure and AT1R overexpression in the cardiomyocyte identifies CM CRP4 as novel anti-hypertrophic and anti-fibrotic factor acting downstream of cGMP/cGKI.

